# Botulinum Toxin as Targeted Neuromodulation in Complex Regional Pain Syndrome: An Anatomy-Informed Mechanistic Review

**DOI:** 10.3390/toxins18040160

**Published:** 2026-03-27

**Authors:** Areerat Suputtitada

**Affiliations:** 1 Department of Rehabilitation Medicine, Faculty of Medicine, Chulalongkorn University, Bangkok 10330, Thailand; prof.areerat@gmail.com; Tel.: +66-81-488-8549; 2 Principles and Practice of Clinical Research (PPCR) Program, Harvard T.H. Chan School of Public Health, Harvard University, Boston, MA 02115, USA

**Keywords:** complex regional pain syndrome, botulinum toxin, neuromodulation, nociplastic pain, sympathetic nervous system, pain phenotyping

## Abstract

Complex regional pain syndrome (CRPS) is a heterogeneous and disabling chronic pain condition characterized by maladaptive neuroplasticity involving persistent peripheral nociceptive input, autonomic dysregulation, and central sensitization. Despite increasing clinical use, the role of botulinum toxin in CRPS remains controversial, with inconsistent outcomes reported across studies. This review synthesizes mechanistic, translational, and clinical evidence suggesting that these apparent inconsistencies may be partly explained by heterogeneity in anatomical targeting and route of administration rather than absence of biological efficacy. Available evidence suggests that botulinum toxin may exhibit its most consistent therapeutic signal when delivered to neural structures directly implicated in dominant CRPS pathophysiology, particularly the sympathetic nervous system and proximal somatic afferents, whereas superficial or non-specific delivery strategies appear to yield more variable responses. Importantly, differences across anatomical targets should not be interpreted as evidence of comparative effectiveness, as observed variation may reflect phenotype selection, procedural heterogeneity, confounding, and differences in outcome reporting. By integrating experimental data, randomized trials, and case-based clinical evidence, an anatomy-informed, route-specific neuromodulation framework is proposed to reconcile existing findings and inform future research. This mechanism-informed perspective is intended to guide rational trial design and phenotype-aligned clinical application of botulinum toxin in CRPS, rather than to provide a definitive evidence-closing synthesis.

## 1. Introduction

Complex regional pain syndrome (CRPS) is a chronic, disabling pain disorder characterized by persistent regional pain disproportionate to the inciting event and accompanied by sensory, autonomic, motor, and trophic abnormalities [1]. The condition most commonly follows limb trauma, fracture, or surgery and is associated with substantial long-term disability, psychological distress, impaired quality of life, and socioeconomic burden [2,3,4]. Despite increasing recognition of its clinical impact, CRPS remains therapeutically challenging, and a considerable proportion of patients develop persistent, refractory symptoms despite multimodal care. CRPS is increasingly understood as a disorder of maladaptive peripheral–central nervous system interaction, in which regionally expressed symptoms reflect distributed neuroimmune, autonomic, and sensory network dysregulation.

CRPS is conventionally classified into type I, in which no definable nerve injury is identified, and type II, in which a distinct peripheral nerve lesion is present [1]. Although diagnostically useful, this binary classification offers limited guidance for mechanism-based treatment selection. Recent quantitative sensory testing meta-analytic data demonstrate that CRPS I versus II, limb location, and sex are associated with broadly comparable sensory profiles, suggesting shared underlying mechanisms across these categories [2]. By contrast, warm versus cold phenotypes exhibit distinct sensory signatures, with heat hyperalgesia predominating in warm CRPS and thermal and mechanical sensory loss characterizing cold CRPS [2]. These findings support stratification by clinical phenotype (and putative dominant mechanisms) rather than by traditional diagnostic labels when interpreting treatment response.

Phenotype-related variables were therefore considered during the evidence synthesis. Where reported in the original studies, we extracted or documented clinical features relevant to CRPS phenotype, including warm versus cold presentation, sensory phenotype descriptors, the presence of motor features such as dystonia or abnormal posturing, and autonomic manifestations including vasomotor or sudomotor changes. Because reporting of these variables was inconsistent across studies, phenotype information was used primarily to contextualize route-specific responses and support mechanistic interpretation, rather than to perform formal subgroup comparisons [2,5,6,7,8,9].

Recent syntheses and guideline discussions have increasingly emphasized the heterogeneity of CRPS and the importance of mechanism-informed treatment strategies. Contemporary clinical guidelines highlight the need for multimodal and individualized approaches to CRPS management [5,9,10,11,12], while several focused reviews have examined the potential role of botulinum toxin in neuropathic pain and related disorders [13]. In parallel, emerging literature has underscored the relevance of phenotype-based stratification, including distinctions such as warm versus cold CRPS, when interpreting treatment responses [2]. The present review builds on these developments by integrating anatomical targeting and route of administration into a mechanism-oriented framework for interpreting the heterogeneous clinical findings reported for botulinum toxin in CRPS.

Mechanistic studies further support the concept of CRPS as a disorder of maladaptive neuroplasticity involving variable contributions of peripheral nociceptive drive, sympathetic–sensory coupling, neurogenic inflammation, and central sensitization [5,6,7,8,9]. The relative dominance of these processes differs across individuals and over time, contributing to the marked heterogeneity observed in clinical presentation and treatment response. This heterogeneity has direct implications for focal neuromodulator interventions, in which therapeutic effects depend on anatomically specific engagement of the dominant pathophysiologic substrate.

Current management strategies for CRPS are multimodal and largely empirical. Pharmacological therapies, including anticonvulsants, antidepressants, corticosteroids, bisphosphonates, and opioids, often provide incomplete or transient benefit and are frequently limited by adverse effects, particularly with long-term use [5,6,7,8,9]. Interventional approaches such as sympathetic nerve blocks, spinal cord stimulation, and dorsal root ganglion stimulation are increasingly employed, yet outcomes vary widely and remain incompletely standardized across centers [11,12]. Consequently, there is ongoing interest in targeted neuromodulator strategies capable of addressing specific drivers of CRPS pathophysiology.

Botulinum toxin has emerged as a potential neuromodulator intervention in CRPS. Beyond its established effects on neuromuscular transmission, botulinum toxin inhibits the release of excitatory neurotransmitters and neuropeptides, including substance P, glutamate, and calcitonin gene-related peptide, modulates peripheral nerve excitability, and attenuates central sensitization [13,14,15]. These actions align closely with mechanisms implicated in CRPS. However, clinical studies evaluating botulinum toxin in CRPS have reported heterogeneous outcomes, limiting its integration into routine practice [16,17,18,19,20,21,22,23,24,25,26,27,28]. Several studies have reported neutral or inconsistent results, highlighting that treatment outcomes may depend on injection depth, anatomical target, and underlying patient phenotype. This apparent inconsistency may be partly explained by heterogeneity in anatomical targeting and route of administration, which determine whether toxin delivery engages sympathetic–sensory coupling, proximal afferent input, or other candidate mechanisms, rather than by absence of biological plausibility. Accordingly, an anatomy-informed, route-specific synthesis is needed to interpret existing findings and guide rational trial design and clinical implementation.

## 2. Results

The available evidence comprises a limited but heterogeneous body of primary clinical studies, including randomized controlled trials, non-randomized interventional investigations, observational cohorts, and detailed case-based reports. Substantial heterogeneity exists with respect to CRPS phenotype, anatomical site, route of administration, dosing strategy, outcome measures, and duration of follow-up. When synthesized according to anatomical target and neuromodulator mechanism rather than study design alone, a coherent pattern of response emerges (Table 1). The most consistent therapeutic signal was observed with delivery targeting the sympathetic ganglia, whereas plexus-level, intramuscular, and superficial approaches demonstrated phenotype-dependent and variable responses. Methodological certainty was highest for randomized studies targeting the sympathetic ganglia, whereas non-randomized and observational evidence was predominantly limited by confounding and selection bias (Supplementary Tables S1–S4). Where reported in the original studies, additional descriptive outcome details, including duration of analgesic effect, follow-up interval, and autonomic outcomes such as temperature change, are summarized in the text below to facilitate interpretation of treatment responses.

### 2.1. Sympathetic Nervous System-Targeted Delivery

Across all routes examined, the most consistent therapeutic signal was observed when botulinum toxin was delivered to the sympathetic nervous system. In a double-blind crossover trial, adjunctive administration of botulinum toxin type A during lumbar sympathetic block significantly prolonged analgesia compared with local anesthetic alone in patients with lower-limb CRPS [16]. The duration of pain relief exceeded the expected pharmacological action of the local anesthetic, supporting a neuromodulator rather than transient blocking effect. In several reports, analgesic benefit persisted beyond the expected duration of local anesthetic blockade, suggesting a neuromodulatory mechanism rather than a transient anesthetic effect. These findings were reinforced by a randomized double-blind controlled trial demonstrating sustained reductions in pain intensity, increases in skin temperature, and improvement in cold intolerance for up to three months following lumbar sympathetic ganglion block augmented with botulinum toxin [17]. Follow-up assessments in these trials extended from several weeks to approximately three months, allowing evaluation of both early analgesic response and short-term durability of effect.

A randomized comparative study further suggested potential serotype-specific differences, reporting that both botulinum toxin type A and type B prolonged sympathetic blockade, with type B associated with a longer duration of analgesia [18]. Although limited by sample size and non-randomized allocation, this observation raises mechanistic questions regarding differential modulation of autonomic neurotransmission.

### 2.2. Plexus-Level and Perineural Neuromodulation

Evidence for brachial plexus-targeted and perineural neuromodulation is currently limited to case-based reports involving upper-limb CRPS. These reports consistently describe rapid attenuation of severe allodynia and functional improvement extending beyond the expected duration of local anesthesia following ultrasound-guided plexus-level or perineural delivery of botulinum toxin [19,20]. Although causal inference cannot be established from such data, the temporal association between intervention and response, together with biological plausibility, supports further investigation of plexus-level targeting in neuropathic-predominant CRPS phenotypes.

### 2.3. Intramuscular Delivery in Motor-Dominant CRPS

Intramuscular botulinum toxin has been primarily applied in CRPS associated with dystonia, spasm, or abnormal posturing. Observational series report moderate reductions in pain and improvements in motor function following electromyography-guided injections [21,22,23,24]. Follow-up periods in these series ranged from several weeks to several months, although outcome reporting was heterogeneous and frequently based on patient-reported measures. Methodological quality is limited by the absence of control groups and reliance on self-reported outcomes, yet the consistency of benefit in motor-dominant presentations suggests a phenotype-specific therapeutic role.

### 2.4. Superficial and Peripheral Tissue Delivery

Cutaneous and subcutaneous delivery strategies yield the least consistent results. A randomized double-blind pilot trial reported no significant analgesic benefit and poor tolerability due to injection-related discomfort [26]. In contrast, case-based evidence describes improvement in selected patients receiving subcutaneous injections [25,27]. These divergent outcomes likely reflect differences in disease phenotype, injection depth, and target engagement. Differences in outcome reporting and follow-up duration across studies further contribute to variability in the observed therapeutic response.

### 2.5. Intra-Articular Delivery

Intra-articular administration has been reported in isolated cases. A case report of glenohumeral injection demonstrated reduction in pain and improvement in range of motion, without significant effects on autonomic or trophic changes [28]. This pattern suggests a localized antinociceptive effect within the joint microenvironment rather than modulation of broader neurovascular or autonomic dysfunction.

### 2.6. Procedural Anatomy and Safety Considerations

Ultrasound-based anatomical studies demonstrate that the spatial relationship between the brachial plexus and pleura varies with patient positioning. Quantitative measurements indicate that lateral decubitus positioning increases the pleura–inferior trunk distance during supraclavicular brachial plexus access, potentially reducing pneumothorax risk [27,28]. These findings derive from anatomical and imaging studies rather than outcome-based clinical trials but represent an important procedural refinement that may enable safer translation of plexus-level neuromodulation.

## 3. Discussion

**Figure 1:**
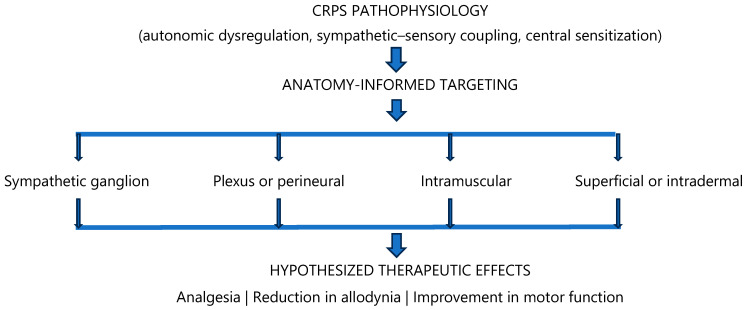
Conceptual framework of anatomy-informed neuromodulation in complex regional pain syndrome (CRPS). The schematic illustrates the proposed relationship between major CRPS pathophysiological mechanisms and potential anatomical targets for botulinum toxin-mediated neuromodulation. Different injection routes may influence distinct neural or peripheral pathways, potentially contributing to heterogeneous clinical responses. The relationships illustrated represent hypothesis-generating conceptual links derived from the current literature rather than definitive mechanistic proof.

This review suggests that the apparent heterogeneity in reported outcomes of botulinum toxin therapy in complex regional pain syndrome may be partly explained by differences in anatomical targeting and route of administration rather than by limited biological efficacy alone. Available studies suggest that botulinum toxin may show its most consistent therapeutic signal in existing studies when delivered to neural structures directly implicated in dominant CRPS pathophysiology, particularly the sympathetic nervous system and proximal somatic afferents, whereas superficial or non-specific delivery strategies yield variable and often limited benefit [16,17,18,19,20,25,26,27]. When the available studies are synthesized according to neuromodulator mechanism and anatomical target, a coherent and biologically plausible pattern of response emerges, with the most consistent therapeutic effects observed following sympathetic ganglion-targeted delivery (Table 1). By contrast, plexus-level, intramuscular, and superficial approaches show variable and phenotype-dependent responses, reflecting differential engagement with dominant pathophysiological drivers of the disorder. An anatomy-informed framework may therefore help reconcile previously conflicting findings and highlights the importance of aligning route of administration with underlying mechanisms of sympathetic sensory coupling, peripheral afferent drive, and maladaptive neuroplasticity in complex regional pain syndrome (Table 2). This conceptual relationship between CRPS pathophysiology, anatomical neuromodulation targets, and hypothesized clinical effects is illustrated in Figure 1.

These findings align with contemporary understanding of CRPS as a disorder of maladaptive neuroplasticity rather than a unitary disease entity. Autonomic dysregulation, sympathetic sensory coupling, neurogenic inflammation, and central sensitization contribute to pain maintenance to varying degrees across individuals [5,9,29]. Importantly, recent quantitative sensory testing meta-analytic data indicate that traditional diagnostic distinctions, such as CRPS type I versus type II or limb location, do not reliably stratify sensory profiles or underlying mechanisms [2]. In contrast, phenotype-based distinctions, particularly warm versus cold CRPS, reveal meaningful mechanistic differences that may influence treatment responsiveness [2]. These observations underscore the limitations of diagnosis driven therapeutic algorithms and highlight the need for phenotype informed neuromodulator strategies.

The observed differences in clinical outcomes across studies should therefore be interpreted cautiously. While anatomical targeting may represent an important explanatory framework for the heterogeneity of responses, it should be considered a hypothesis generating interpretation rather than a definitive causal explanation, as multiple factors such as phenotype variability, procedural differences, dosing strategies, and outcome reporting may also contribute to the observed variability.

Sympathetic ganglion-targeted delivery represents the most robustly supported application of botulinum toxin in CRPS. Randomized trials demonstrate that adjunctive administration during sympathetic block prolongs analgesia and produces sustained autonomic changes beyond the expected duration of local anesthetics [16,17,18]. These findings support a central role for sympathetic sensory coupling in maintaining pain and autonomic disturbance in a subset of patients. Experimental and clinical data indicate that abnormal sympathetic efferent activity can sensitize primary afferent nociceptors and amplify peripheral input to the central nervous system [6,9,30]. The observation of potential serotype-specific differences further suggests that botulinum toxin formulations may differentially modulate autonomic neurotransmission, an area warranting further investigation [18].

Plexus-level and perineural neuromodulation, although supported primarily by case-based evidence, offers important mechanistic insight. In refractory upper-limb CRPS, particularly neuropathic-predominant phenotypes, proximal modulation of somatic afferent transmission may interrupt ongoing peripheral drivers of central sensitization [19,20]. This interpretation is consistent with experimental models demonstrating that sustained peripheral afferent input can maintain central hyperexcitability even after the inciting injury has resolved [7,8]. Procedural refinements informed by ultrasound anatomy, including lateral decubitus positioning to increase the pleura–plexus distance, may enhance the safety and feasibility of such approaches and facilitate their evaluation in prospective studies [29,31].

Intramuscular botulinum toxin occupies a distinct niche in motor-dominant CRPS presentations characterized by dystonia or abnormal posturing. In these cases, abnormal muscle activation and altered spindle input may serve as ongoing sources of nociceptive afferent drive. By reducing aberrant motor output and afferent feedback, intramuscular delivery may indirectly modulate pain processing [21,22,23,24]. However, the absence of controlled trials limits generalizability, and this approach should be considered phenotype-specific rather than broadly analgesic.

Several limitations of the current evidence base warrant consideration. High-quality randomized data remain scarce, outcome measures are heterogeneous, and phenotypic stratification is inconsistently reported. Procedural variables, including dose, dilution, injection technique, and imaging guidance, are often inadequately described. Where available, additional study and procedural details were incorporated into the manuscript text and study summaries; however, several primary reports did not consistently provide complete demographic or dosing information. Furthermore, several studies lacked detailed reporting of technical parameters such as toxin dilution, injection volume, and standardized injection protocols, which limits the ability to directly compare technical approaches across studies. These limitations preclude quantitative synthesis and highlight the need for hypothesis-driven rather than empiric application of botulinum toxin in CRPS [30,32,33,34,35].

Phenotype characteristics were inconsistently reported across the included studies, limiting the ability to draw definitive conclusions regarding phenotype-specific treatment effects. Accordingly, any apparent phenotype-dependent responses observed in the current synthesis should be interpreted as hypothesis-generating rather than confirmatory. Future studies incorporating prospective phenotype stratification may help clarify whether specific CRPS subtypes respond preferentially to particular neuromodulation targets.

Despite these constraints, the available evidence supports reconceptualizing botulinum toxin therapy in CRPS as a form of precision neuromodulation. Aligning anatomical targeting with dominant pathophysiological mechanisms reconciles conflicting findings in the literature and provides a rational framework for future investigation. Prospective studies should prioritize phenotype-based stratification, directly compare autonomic versus somatic neural targets, incorporate autonomic and functional outcome measures, and integrate procedural safety optimization. Such an approach may facilitate the transition of botulinum toxin therapy in CRPS from empirical use toward mechanism-informed neuromodulation in a condition characterized by profound heterogeneity and unmet clinical need.

## 4. Conclusions

Botulinum toxin may represent a neuromodulator option for selected complex regional pain syndrome phenotypes when delivered to anatomically and mechanistically relevant targets. The available evidence suggests that therapeutic response may depend on the alignment between anatomical target, route of administration, and the dominant underlying pathophysiology. Sympathetic ganglion-targeted delivery demonstrates the most consistent signal, while plexus-level, intramuscular, and superficial approaches appear more variable and potentially phenotype-dependent, with current evidence largely remaining hypothesis-generating.

An anatomy-informed, route-specific framework may help reconcile conflicting findings in the existing literature and provides a rational foundation for future clinical trials and translational neuromodulation strategies. Progress in this field will depend on rigorous phenotypic stratification, mechanistically informed target selection, and careful procedural standardization and safety optimization. Such an approach may facilitate a transition of botulinum toxin therapy in CRPS beyond empirical use toward precision neuromodulation in a condition characterized by profound heterogeneity and unmet clinical need.

## 5. Materials and Methods

From each eligible study, data were extracted on CRPS subtype, anatomical site, route of botulinum toxin administration, target structure, toxin formulation and dose, comparator (if applicable), clinical outcomes, and duration of follow-up. Data extraction was performed using a predefined framework focused on anatomical and mechanistic relevance rather than study design alone.

The literature search was conducted in PubMed/MEDLINE, Embase, and Scopus from database inception to November 2025. The search employed combinations of the following terms: complex regional pain syndrome, CRPS, botulinum toxin, botulinum toxin type A, botulinum toxin type B, sympathetic block, brachial plexus, perineural, intramuscular, and intradermal. Boolean operators were used to combine search terms, and the full database-specific search strategies are provided in the Supplementary Materials (Table S5). Reference lists of relevant articles were also screened to identify additional eligible studies.

Study screening and eligibility assessment were performed by the author using predefined eligibility criteria. Titles and abstracts were initially screened to identify potentially relevant studies, followed by full-text evaluation of eligible articles. The study identification and selection process is summarized in a flow diagram provided in the Supplementary Materials (Figure S1).

Eligible studies included randomized controlled trials, non-randomized interventional studies, observational cohorts, case series, and case reports involving human participants diagnosed with CRPS. Systematic reviews, meta-analyses, editorials, conference abstracts, and animal studies were excluded. No language restrictions were applied.

Given substantial heterogeneity in study design, patient populations, anatomical targets, and outcome measures, a qualitative narrative synthesis was performed rather than a quantitative meta-analysis. This approach was selected to preserve mechanistic interpretability across heterogeneous pain phenotypes.

Studies were categorized a priori according to their primary anatomical neuromodulator target, rather than by study design alone, to reflect underlying pathophysiological mechanisms relevant to CRPS. Four operational categories were defined: sympathetic ganglion targeting, plexus or perineural targeting, intramuscular delivery, and superficial or peripheral tissue delivery.

Methodological quality was assessed using tools appropriate to study design. Randomized controlled trials were evaluated using the Cochrane Risk of Bias 2 (ROB 2, version 2019) tool. Non-randomized interventional studies were assessed using ROBINS-I (version 2016). Observational cohorts and case series were appraised using the Newcastle–Ottawa Scale (NOS). Case reports were evaluated qualitatively for diagnostic clarity, transparency of intervention description, outcome reporting, and biological plausibility. Risk-of-bias assessments were used to inform the interpretive context of the qualitative synthesis rather than to quantitatively weight individual studies.

To further contextualize the overall strength of the evidence base, certainty of evidence was described using an adapted qualitative application of the GRADE framework. Given the heterogeneity in study designs and outcome measures, GRADE ratings were applied descriptively to summarize relative confidence in the available evidence rather than as a formal basis for clinical recommendation.

## Figures and Tables

**Table 1:** Clinical studies of botulinum toxin in complex regional pain syndrome grouped by anatomical target.

Anatomical Target	Study (Ref)	Study Design (n)	CRPS Site/Phenotype	BoNT Formulation/Dose/Route/Guidance	Follow-Up	Principal Findings
**Sympathetic ganglion**	Carroll et al. [16]	Randomized, double-blind, controlled crossover trial (n = 9)	Lower-limb CRPS	BoNT-A 75 U + bupivacaine; lumbar sympathetic block; fluoroscopic guidance	Median time to analgesic failure: 71 days (95% CI 12–253)	Prolonged analgesia compared with local anesthetic alone
	Yoo et al. [17]	Randomized, double-blind controlled trial (n = 47)	Lower-limb CRPS, predominantly cold phenotype	BoNT-A 75 U + levobupivacaine (8 mL); lumbar sympathetic ganglion block; fluoroscopic guidance	1 and 3 months	Sustained pain reduction, increased skin temperature, and improved cold intolerance
	Lee et al. [18]	Randomized, double-blind comparative study (n = 18; 9 per group)	Lower-limb CRPS	BoNT-A 100 U or BoNT-B 5000 U; lumbar sympathetic block; fluoroscopic guidance	up to 8 weeks (56 days)	Both serotypes prolonged analgesia, with longer duration observed in the BoNT-B group
**Plexus or perineural**	Moon et al. [19]	Case series (n = 7)	Neuropathic pain including CRPS	BoNT-A; ultrasound-guided nerve block	not clearly specified	Analgesia exceeding expected duration of nerve block
	Meyer-Frießem et al. [20]	Case series (n = 60)	Peripheral neuropathic pain (including CRPS subset)	BoNT-A; perineural injection; ultrasound-guided	≥7 days to several months	Mean reduction: 24.8% Responders: 57% Responders’ reduction: 43.4% No sensory deficit (QST unchanged)
**Intramuscular**	Fallatah [21]	Case report (n = 1)	Upper-limb CRPS	Interscalene brachial plexus block (bupivacaine); ultrasound-guided; adjunct BoNT-A trigger point injection	up to 3 months	Rapid and complete pain relief with functional recovery; sustained improvement over follow-up
	Cordivari et al. [22]	Case series (n = 14; CRPS subset n = 4)	Dystonia-associated CRPS and other movement disorders	AbobotulinumtoxinA 450–1200 U; EMG-guided intramuscular injection (lumbricals and forearm muscles)	NR	Pain reduction and muscle relaxation; functional improvement in selected cases
	Kharkar et al. [23]	Case series (n = 37)	CRPS with dystonia (motor-dominant phenotype)	BoNT-A (dose individualized); intramuscular; EMG-guided	~4 weeks	Reduction in pain and dystonia with functional improvement
	Schilder et al. [24]	Prospective experimental study (n = 17)	CRPS with tonic dystonia	OnabotulinumtoxinA 20 U; intramuscular injection (extensor digitorum brevis)	2 weeks	Normal BoNT-A responsiveness (CMAP reduction > 20%); slightly reduced effect vs controls
**Superficial or peripheral tissue**	Lessard et al. [25]	Case series (n = 20)	Upper-limb CRPS (refractory to sympathetic blocks)	OnabotulinumtoxinA < 100 U/session; subcutaneous grid injection (10 IU/cm^2^; max 100 IU/session)	Monthly repeated sessions (mean 8.85)	Mean VAS reduction 2.05 (~22.9%); significant improvement (*p* < 0.01); cumulative effect over repeated treatments
	Safarpour et al. [26]	Pilot randomized double-blind controlled study (n = 14; RCT n = 8 + open-label n = 6)	CRPS with allodynia	BoNT-A; intradermal and subcutaneous injection (5 U/site; total 40–200 U)	3 weeks–2 months	No significant analgesic benefit; poor tolerability
	Tereshko et al. [27]	Case report (n = 1)	Upper-limb CRPS type 1	BoNT-A 50–80 IU; subcutaneous multi-site injection (hand and forearm; non–image-guided)	Up to ~2–3 months	Repeated injections improved pain, allodynia, and motor function with no adverse effects
**Intraarticular**	Bellon et al. [28]	Case report (n = 1)	CRPS (upper limb; shoulder involvement)	BoNT-A 100 U; intra-articular injection (glenohumeral joint)	Up to 4 months	Pain reduction and improved ROM; no effect on autonomic or trophic changes

**Abbreviations:** CRPS, complex regional pain syndrome; BoNT-A, botulinum toxin type A; EMG, electromyography; NR, not reported. Findings are descriptive and reflect within-study outcomes rather than pooled effect estimates. Reported results should not be interpreted as comparative efficacy across anatomical targets.

**Table 2:** Hypothesized mechanistic mapping of route-specific effects of botulinum toxin in complex regional pain syndrome and the corresponding evidence context.

Anatomical Target	Primary Neuromodulatory Mechanism	Dominant Pathophysiological Process Addressed	Expected Clinical Effect	Evidence Context
**Sympathetic ganglion**	Inhibition of cholinergic sympathetic transmission and downstream autonomic neurotransmitter release	Sympathetic–sensory coupling, vasomotor instability, and cold-type autonomic dysregulation	Sustained analgesia with improvement in vasomotor symptoms and skin temperature asymmetry	Randomized controlled and comparative studies [16,17,18]
**Plexus or perineural**	Attenuation of proximal nociceptive afferent signaling and ectopic peripheral input	Persistent peripheral afferent drive sustaining central sensitization and mechanical allodynia	Rapid pain relief, reduction in allodynia, and functional improvement beyond expected anesthetic duration	Case series and clinical reports [19,20]
**Intramuscular**	Reduction of abnormal muscle overactivity and spindle-related afferent discharge	Motor-driven nociceptive input in dystonia-associated or spasm-predominant CRPS	Improvement in pain, dystonia, abnormal posturing, and functional outcomes	Observational series and mechanistic studies [21,22,23,24]
**Superficial or intradermal/subcutaneous**	Modulation of superficial nociceptor activity and neurogenic inflammatory mediators	Cutaneous hypersensitivity, allodynia, and heterogeneous peripheral sensitization	Variable analgesic response; may improve selected phenotypes but limited or poorly tolerated in severe allodynia	Pilot randomized studies, case series, and case reports [25,26,27]
**Intra-articular**	Local inhibition of nociceptive mediator release within the joint microenvironment	Joint-related peripheral nociceptive input contributing to pain and restricted mobility	Reduction in pain and improved range of motion, with minimal effect on autonomic or trophic changes	Case-based evidence [28]

This framework links anatomical targeting with dominant pathophysiological mechanisms relevant to CRPS and is intended to inform mechanism-guided trial design and clinical application. The relationships presented in this framework should be interpreted as hypothesis-generating conceptual links derived from existing literature rather than definitive mechanistic proof.

## Data Availability

No new data were created or analyzed in this study. Data sharing is not applicable to this article.

## Supplementary Materials

The following supporting information can be downloaded at: https://www.mdpi.com/article/10.3390/toxins18040160/s1. Figure S1: PRISMA 2020 flow diagram illustrating study identification, screening, eligibility assessment, and inclusion of studies evaluating botulinum toxin interventions in complex regional pain syndrome. Table S1: Risk of Bias 2 (ROB 2) assessment for randomized controlled trials. Table S2: ROBINS-I assessment for non-randomized interventional studies. Table S3: Newcastle–Ottawa Scale (NOS) assessment for observational studies. Table S4: GRADE certainty-of-evidence summary. Table S5: Database search strategies used for literature identification.

## Abbreviations

The following abbreviations are used in this manuscript:

BoNT/A	Botulinum toxin type A
BoNT/B	Botulinum toxin type B
CRPS	Complex regional pain syndrome
EMG	Electromyography
GRADE	Grading of Recommendations Assessment, Development and Evaluation
NOS	Newcastle–Ottawa Scale
ROB 2	Risk of Bias 2
ROBINS-I	Risk of Bias In Non-randomized Studies of Interventions
