# Supplementary material for: Botulinum Toxin as Targeted Neuromodulation in Complex Regional Pain Syndrome: An Anatomy-Informed Mechanistic Review
Source: Toxins (Basel). 2026 Mar 27;18(4):160. doi: 10.3390/toxins18040160 (PMC13119950; doi:10.3390/toxins18040160)
Supplement: Supplementary file 1 [file toxins-18-00160-s001.zip › toxins-4186906-supplementary.pdf]

Supplementary Materials: Botulinum Toxin as Targeted Neuromodulation in Complex Regional Pain Syndrome: An Anatomy-Informed Mechanistic Review

Areerat Suputtitada [1,2]

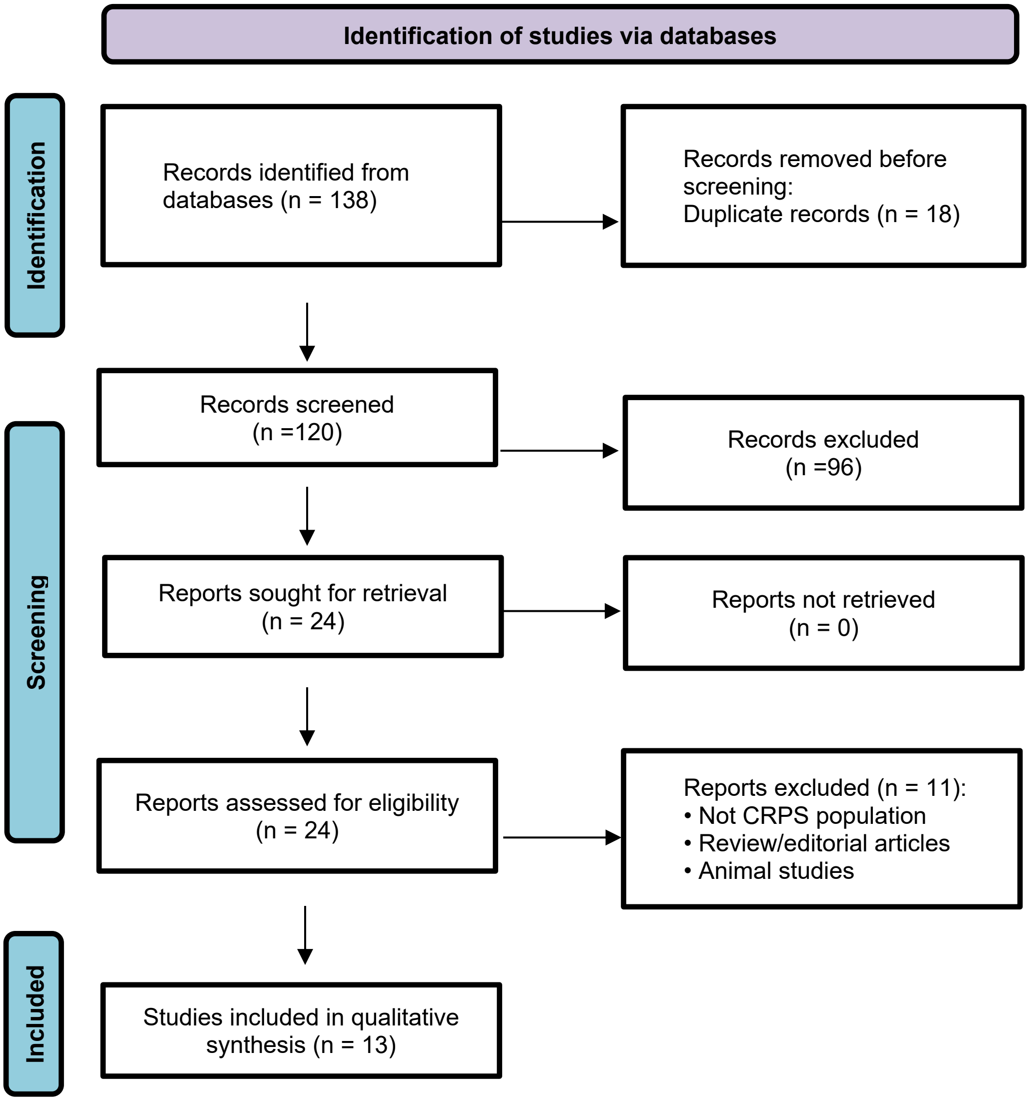

Figure S1. PRISMA 2020 flow diagram illustrating study identification, screening, eligibility assessment, and inclusion of studies evaluating botulinum toxin interventions in complex regional pain syndrome.

Table S1. Risk of Bias 2 (ROB 2) assessment for randomised controlled trials

Study	Randomisation	Deviations	Missing data	Outcome measurement	Selective reporting	Overall risk
Carroll et al [16].	Some concerns	Low	Low	Some concerns	Low	Some concerns
Yoo et al [17].	Low	Low	Low	Low	Low	Low
Lee et al [18].	Some concerns	Some concerns	Low	Some concerns	Some concerns	Some concerns
Safarpour et al [26].	Low	Low	Low	Some concerns	Some concerns	Some concerns

Risk of bias was assessed using the Cochrane Risk of Bias 2 tool across five domains: randomisation process, deviations from intended interventions, missing outcome data, measurement of outcomes, and selection of the reported result. Overall risk reflects the highest level of concern across domains.

Table S2. ROBINS-I assessment for non-randomised interventional studies

Study	Confounding	Selection	Classification	Deviations	Missing data	Outcome measurement	Reporting	Overall risk
Moon et al [19].	Serious	Moderate	Low	Moderate	Low	Moderate	Moderate	Serious
Meyer-Frießem et al [20].	Serious	Moderate	Low	Moderate	Low	Moderate	Moderate	Serious
Fallatah [21].	Serious	Serious	Low	Low	Low	Moderate	Serious	Serious
Tereshko et al [27].	Serious	Moderate	Low	Low	Low	Moderate	Moderate	Serious

Risk of bias was assessed using the ROBINS-I tool across seven domains. Overall risk reflects the highest level of bias identified and indicates methodological certainty rather than magnitude of clinical effect.

Table S3. Newcastle–Ottawa Scale (NOS) for observational studies

Study	Selection	Comparability	Outcome	Total
Cordivari et al [22]	★★☆☆	☆☆	★★☆	★★★★☆☆☆☆☆
Kharkar et al [23]	★★★☆	☆☆	★★☆	★★★★★☆☆☆
Schilder et al [24]	★★☆☆	☆☆	★★☆	★★★★☆☆☆☆☆
Lessard et al [25]	★★★☆	☆☆	★★☆	★★★★★☆☆☆
Bellon et al [28]	★★☆☆	☆☆	★★☆	★★★★★☆☆☆

NOS quality classification:

- Very good quality: 9–10 stars
- Good quality: 7–8 stars
- Satisfactory quality: 5–6 stars
- Unsatisfactory quality: 0–4 stars

Studies were assessed across selection, comparability, and outcome domains using the Newcastle Ottawa Scale. Stars indicate methodological quality, with a maximum of nine stars. No study received comparability stars due to absence of control groups, limiting causal inference and increasing susceptibility to confounding.

Table S4. GRADE certainty of evidence summary

Outcome	Key studies (refs)	Study design	Risk of bias	Inconsistency	Indirectness	Imprecision	Publication bias	Overall certainty
Pain reduction with sympathetic ganglion targeting	Carroll et al [16]; Yoo et al [17]; Lee et al [18].	Randomised controlled trials	Not serious	Not serious	Not serious	Serious	Undetected	Moderate
Pain reduction with plexus or perineural targeting	Moon et al [19]; Meyer-Frießem et al [20]; Fallatah [21].	Observational studies (case series and uncontrolled studies)	Serious	Serious	Not serious	Serious	Suspected	Low
Motor improvement in motor-dominant CRPS with intramuscular injection	Cordivari et al [22]; Kharkar et al [23]; Schilder et al [24].	Observational studies (case series and uncontrolled experimental study)	Serious	Serious	Serious (indirectness present)	Serious	Suspected	Low
Analgesic effect of superficial or peripheral tissue delivery	Lessard et al [25]; Safarpour et al [26]; Tereshko et al [27]; Bellon et al [28].	Randomized controlled trial and observational studies (cohort, case series, and case reports)	Not serious to serious	Not serious to serious	Not serious to serious	Serious	Suspected	Low to very low

Certainty of evidence was assessed using the Grading of Recommendations Assessment Development and Evaluation framework. Outcomes were downgraded for risk of bias, inconsistency, indirectness, imprecision, and suspected publication bias. Certainty ratings reflect confidence in the estimated effect rather than the magnitude or clinical importance of the effect.

Table S5. Database Search Strategies Used for Literature Identification

Database	Search Strategy
**PubMed / MEDLINE**	("complex regional pain syndrome"[Title/Abstract] OR CRPS[Title/Abstract]) AND ("botulinum toxin"[Title/Abstract] OR "botulinum toxin type A"[Title/Abstract] OR "botulinum toxin type B"[Title/Abstract]) AND (sympathetic[Title/Abstract] OR "sympathetic block"[Title/Abstract] OR "brachial plexus"[Title/Abstract] OR perineural[Title/Abstract] OR intramuscular[Title/Abstract] OR intradermal[Title/Abstract])
**Embase**	('complex regional pain syndrome' OR CRPS) AND ('botulinum toxin' OR 'botulinum toxin type A' OR 'botulinum toxin type B') AND (sympathetic OR 'sympathetic block' OR 'brachial plexus' OR perineural OR intramuscular OR intradermal)
**Scopus**	TITLE-ABS-KEY ("complex regional pain syndrome" OR CRPS) AND TITLE-ABS-KEY ("botulinum toxin" OR "botulinum toxin type A" OR "botulinum toxin type B") AND TITLE-ABS-KEY (sympathetic OR "sympathetic block" OR "brachial plexus" OR perineural OR intramuscular OR intradermal)
